# Bleeding in Tetanus

**Published:** 1881-06

**Authors:** 


### BLEEDING IN TETANUS.

The following heroic treatment is given by a correspondent of the *Druggists' Circular*, February, 1881:—

Peter Forbes, a colored man employed by a farmer, cut one of his fingers very severely. Little notice was taken of the cut at first, but the man was struck with the fact that the long gash in his finger was scarcely bleeding. He said nothing about it until next morning, when he awoke and found the muscles of his mouth all contracted, and felt unable to open it sufficiently to speak. A physician was called in, who, upon arriving, examined him, and told him he could do nothing for him, as his case was a fatal one. Having left the house, he was shortly after sent for a second time, and went through the same course as before, saying he could do nothing for the patient, that there was no cure for his case that he knew of, and then retired. Peter, thinking that he was going to die, thought he would resort to some means himself, hoping to effect a cure. He went to a closet and found an old shoemaker's awl and sharpened it, then he sat down and commenced to draw it across his *tongue*, cutting long gashes in it. As soon as he cut the first gash he felt greatly relieved; shortly afterwards breathing became more easy and the muscles somewhat relaxed. He, finding this to answer so well, repeated the operation several times and effected a *speedy cure*. To-day he is well and hearty and at his work. This was pronounced by several doctors as a case of lockjaw.

